# Trajectory Optimization of Laser-Charged UAVs for Charging Wireless Rechargeable Sensor Networks

**DOI:** 10.3390/s22239215

**Published:** 2022-11-27

**Authors:** Ning Liu, Chuanwen Luo, Jia Cao, Yi Hong, Zhibo Chen

**Affiliations:** 1 School of Information Science and Technology, Beijing Forestry University, Beijing 100083, China; 2 Engineering Research Center for Forestry-Oriented Intelligent Information Processing of National Forestry and Grassland Administration, Beijing 100083, China

**Keywords:** laser-charged UAV, wireless power transfer, mobile unmanned vehicle, wireless rechargeable sensor network, trajectory optimization

## Abstract

This paper considers a laser-powered unmanned aerial vehicle (UAV)-enabled wireless power transfer (WPT) system. In the system, a UAV is dispatched as an energy transmitter to replenish energy for battery-limited sensors in a wireless rechargeable sensor network (WRSN) by transferring radio frequency (RF) signals, and a mobile unmanned vehicle (MUV)-loaded laser transmitter travels on a fixed path to charge the on-board energy-limited UAV when it arrives just below the UAV. Based on the system, we investigate the trajectory optimization of laser-charged UAVs for charging WRSNs (TOLC problem), which aims to optimize the flight trajectories of a UAV and the travel plans of an MUV cooperatively to minimize the total working time of the UAV so that the energy of every sensor is greater than or equal to the threshold. Then, we prove that the problem is NP-hard. To solve the TOLC problem, we first propose the weighted centered minimum coverage (WCMC) algorithm to cluster the sensors and compute the weighted center of each cluster. Based on the WCMC algorithm, we propose the TOLC algorithm (TOLCA) to design the detailed flight trajectory of a UAV and the travel plans of an MUV, which consists of the flight trajectory of a UAV, the hovering points of a UAV with the corresponding hovering times used for the charging sensors, the hovering points of a UAV with the corresponding hovering times used for replenishing energy itself, and the hovering times of a UAV waiting for an MUV. Numerical results are provided to verify that the suggested strategy provides an effective method for supplying wireless rechargeable sensor networks with sustainable energy.

## 1. Introduction

Wireless sensor networks (WSNs) are widely employed in a variety of application scenarios, including environmental monitoring, forest fire prevention, traffic control, and so on [1]. In these networks, sensors can gather data about the environment and perform functions such as processing, storing, and communicating. However, the available energy of the sensors is quite constrained, and replacing or recharging the batteries for them is often difficult, particularly in complex terrain areas. To guarantee their sustainable operation, radio frequency (RF)-based wireless power transfer (WPT) has emerged as a possible solution for a sustainable energy source for sensors. These WSNs are called wireless rechargeable sensor networks (WRSNs).

Traditional energy-supplying methods for WRSNs such as solar or wind are expensive and difficult to maintain and are unreliable. Meanwhile, it is inefficient to install the energy transmitter in a fixed position to charge the sensors since the energy transmitter is too far away from the sensors or the equipment can be easily damaged. UAVs can be used as mobile chargers to replenish the energy for sensors in WRSNs when they are installed in the energy transmitter due to their high maneuverability, deployment flexibility, etc. UAVs can be equipped with RF transmission equipment to charge sensors through line-of-sight (LoS) energy transmission links [2].

Although UAVs have many advantages that can be used for charging the sensors in WRSNs, they cannot work continuously because of the limited airborne energy capability, especially for long-duration and energy-intensive flight missions. With the development of laser-charging technologies, a UAV can have its energy replenished by laser beams transmitted by laser-emitting devices. This is because the laser beams have a high energy density and low beam divergence. However, this will increase the energy consumption and task completion times of a UAV when laser-emitting devices are deployed in fixed locations. To overcome the above problems, an MUV equipped with laser transmitters can be used to charge a UAV due to its mobility and good speed.

In this paper, we consider a novel wireless network architecture, which consists of rechargeable sensors, a UAV, and an MUV. In the architecture, a UAV loaded with a wireless transmitter is used to charge the sensors and an MUV carrying a laser transmitter is applied to replenish the energy of the UAV by transmitting laser beams.

In the architecture, we study the trajectory optimization of laser-charged UAVs for charging WRSNs (TOLC problem), whose objective is to minimize the total working time of a UAV by optimizing the flight trajectory of a UAV and the travel plans of an MUV cooperatively. In the problem, it is simultaneously satisfied that the energy of each sensor in a WRSN is greater than or equal to the threshold and that the remaining energy of a UAV can support it to fly at any instant. The contributions of this paper are summarized below.

(1) We propose a new WRSN assisted by an MUV and a laser-charged UAV. In the network, a UAV is used as a mobile charger to supplement the energy for sensors and an MUV is used to charge a UAV. To the best of our knowledge, this is the first work to study an MUV-assisted laser-charged UAV for charging sensors in a WRSN from the perspective of the combined flight trajectory of a UAV and travel plans of an MUV.

(2) We propose the trajectory optimization of laser-charged UAVs for charging WRSNs (TOLC problem) in an MUV-assisted laser-powered UAV-enabled WRSN. This problem needs to design the detailed flight trajectory of a UAV and the travel plans of an MUV cooperatively, which consist of the flight trajectory of the UAV, the hovering points of the UAV with the corresponding hovering times used for charging the sensors, the hovering points of the UAV with the corresponding hovering times used for replenishing the energy itself, and the hovering times of the UAV when waiting for an MUV. Then, we prove that the problem is NP-hard.

(3) To solve the TOLC problem, we first propose a clustering algorithm WCMC to cluster the sensors and compute the weighted center of each cluster. As far as we know, this is the first method to cluster sensors using a weighted center and minimum covering circle. Based on the WCMC algorithm, we propose the TOLCA algorithm to solve the TOLC problem.

(4) We conduct extensive simulations to illustrate the effectiveness of the proposed algorithm for the TOLC problem.

The remainder of this paper is organized as follows. Section 2 introduces the related works. In Section 3, we introduce some models and definitions of the studied problem. In Section 4, we propose the TOLCA for solving the TOLC problem. The simulations are shown in Section 5. Section 6 concludes this paper.

## 2. Related Works

In this section, we briefly review the related works about the research problem on the following two topics: charging sensors with UAVs and energy-replenishing methods of UAVs.

### 2.1. Charging Sensors with UAVs

Recently, many research problems of UAV-assisted wireless power transmission have been studied, such as [3,4,5,6,7,8,9,10,11], where UAVs were loaded energy transmitters applied to the supply of energy to ground devices.

In [3,4], a simple two-user scenario was taken into account, which maximizes the received energy of two receivers. The findings in [3] demonstrated that if the distance between the two receivers is less than a certain threshold, the UAV must hover at the set position between the two receivers during the entire charging procedure; otherwise, it must hover and fly between the two above the connecting line. The authors in [4] optimized the UAV’s trajectory using Q-learning reinforcement learning to maximize the least acquired energy of the two energy receivers. In [5], a two-user UAV-based WPT system was investigated, in which a heuristic hover-flight trajectory based on symmetric position hovering was proposed to maximize the total received energy of two receivers under the restrictions of the maximum UAV velocity, maximum/minimum altitude, and beam width. In [6], Hu et.al introduced a 1D UAV trajectory design problem, in which a UAV was used to charge a set of ground nodes with a linear topology. The objective of the problem was to maximize the minimal received energy among all the ground nodes. In [7], Yang et al. proposed a genetic algorithm based on successive hover and fly motions to design the trajectory of a UAV for charging users in the charging area. The goal of the problem was to maximize the least received energy for all users under the UAV speed constraint. In [8], Yuan et al. investigated a UAV-assisted WPT network that considered the nonlinear energy-harvesting process. The objective of the problem was to maximize the minimum harvested energy among ground devices with a maximum flight speed restriction. In [9], Yuan et al. introduced a UAV-enabled multi-user WPT network in which both the nonlinear energy-harvesting model and the uniform linear array directional antenna structure were considered to concurrently optimize the UAV trajectory and the orientation of the directional antenna on the UAV to maximize the minimum harvested energy among all users. In [10], Feng et al. investigated an energy-harvesting optimization problem for a UAV-aided WPT system in which the UAV’s three-dimensional position, beam pattern, charging period, and trajectory optimization were considered to maximize the energy gathered by all energy receivers. In [11], Yan et al. studied the use of a UAV as a charger, which considered the power consumption of a UAV during hovering and flying, the link loss during charging from the base station, and the conversion loss of the energy received by the sensors. The objective of this study was to maximize the total obtained energy of all sensors by optimizing the UAV’s deployment.

### 2.2. Energy-Replenishing Methods for UAVs

Due to the limitations of UAV’s airborne energy, UAVs need to have their energy replenished when they perform tasks. Many researchers are devoted to energy-harvesting methods for UAVs, where they are used as mobile data collectors or mobile edge computing servers, such as [12,13,14,15,16,17].

In [12], Suzuki et al. investigated an automated battery replacement system for UAVs, which included a ground station with replaceable batteries to supply UAVs. In [13], Li et al. introduced an energy-efficient cooperative strategy with multiple rechargeable UAVs for supporting seamless information services of ground devices, and the cooperation of multiple UAVs could formulate a closed chain. The goal was to maximize the energy efficiency of the system through joint node assignments and UAV configuration optimization. In [14], Fu et al. proposed a reinforcement learning method to design the path for UAVs to collect sensor data and use fixed charging piles to supplement energy. They divided the physical environment into numerous grids and each grid had a wireless charger in the center for charging the UAV. In [15], Hu et al. studied a wireless-powered UAV-assisted mobile edge computing architecture, in which the UAV was used as the mobile edge computing server and for the energy and information relay of users. In this architecture, a fixed access point was used to charge the UAV through the laser beam transmitters. In [16], Wang et al. considered a laser-powered aerial mobile edge computing system in which the high-altitude-platform UAV transferred laser energy to charge aerial users, and the users offloaded their computation tasks to the high-altitude-platform UAV. In [17], Zhu et al. investigated the UAV-aided data collection problem in a large-scale WSN in which a truck carrying backup batteries moved together with a UAV and the UAV could fly back to the truck for battery replenishment. The goal of the problem was to minimize the total mission time for gathering data from all sensors.

In previous research on charging sensors with UAVs, the UAV was not considered as the replenished energy. In the above research on the energy-replenishing methods of UAVs, the methods of changing batteries using a truck or stations have the disadvantages of high costs and poor flexibility. This is because a truck moving with a UAV may not be able to reach areas with complex terrains, and UAVs traveling to and from stations to change batteries increases the working time. Using fixed access points to charge UAVs increases the working time and reduces the flexibility of UAVs.

To overcome the above disadvantages, we study the trajectory optimization of laser-charged UAVs for charging WRSNs (TOLC problem) in a new MUV-assisted laser-powered UAV-enabled WRSN. In the problem, a UAV is used as a mobile charger to supplement the energy for sensors and an MUV is used to charge the UAV.

## 3. Model and Problem Definition

### 3.1. Network Model

**Figure 1:**
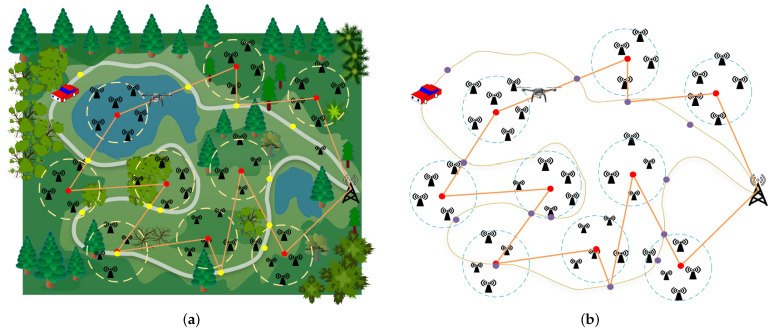
Energy harvesting of a WSN in a field environment.(**a**) System model of a MUV-assisted laser-charged UAV-based WSN; (**b**) Illustration of the path planning of the UAV and MUV in the system.

As shown in Figure 1, we consider a wireless sensor network (WSN), which consists of a base station, an MUV, a rotary-wing UAV *f*, and *n* sensors, that is deployed in a two-dimensional space. Let $S=\{s_{1},s_{2},...,s_{n}\}$ denote the set of *n* sensors, where the coordinates of each sensor $s_{i}\in S$ are denoted by $\left(x_{i},y_{i}\right)$. We use $E_{s}^{i}$ to denote the initial energy of each sensor $s_{i}\in S$ and $E_{s}^{i}<E_{0}$, where $E_{0}$ is the threshold of the sensors’ energy.

We use a rotary-wing UAV as the air-mobile charger for replenishing the energy of the sensors. The UAV, which has an initial energy (capacity) $E_{v}$ and a constant speed $v_{f}$, flies at a fixed altitude *H*. Only when $E_{s}^{i}<E_{0}$ does the sensor $s_{i}$ need to be charged by the UAV. The UAV charges the sensors by transmitting RF signals with power $P_{R}$ when it is hovering. Let *R*(R>H) be the transmitting range of the RF signals of the UAV. Therefore, we can obtain the radius of the coverage disk of the UAV $r=\sqrt{R^{2}-H^{2}}$ when it hovers at altitude *H*. The UAV contains $n_{0}$ antennas and it can charge at most, $n_{0}$ sensors simultaneously, i.e., one antenna can be only used for charging one sensor.

An MUV is used to charge a UAV, which departs from the base station $s_{0}\left(x_{0},y_{0}\right)$ at the same time as the UAV and travels along a fixed path *L*. Let $v_{max}$ be the maximum velocity of the MUV. The MUV can transmit laser radio energy to the UAV when the UAV is hovering over it. Assume the MUV carries enough energy for traveling and for transferring the laser radio. We use $P_{L}$ to denote the laser transmission power of the MUV.

### 3.2. RF Power Transmission Model

In this paper, we use the model of the RF power transmission from the UAV to the sensors proposed in [18], where the wireless channel between the UAV and each sensor is modeled as a Los ground-space channel. Therefore, the channel power gain from the UAV to the sensor $s_{j}$ at hover point $q_{i}$ is modeled as

(1) $$h_{j}^{i}=\frac{\gamma_{0}}{d^{2}\left(q_{i},s_{j}\right)}=\frac{\gamma_{0}}{{\left(x_{q}^{i}-x_{j}\right)}^{2}+{\left(y_{q}^{i}-y_{j}\right)}^{2}+H^{2}},$$

where $\gamma_{0}$ stands for the channel power gain at *d* = 1 m and its value depends on the antenna gain and carrier frequency, and $d(q_{i},s_{j})$ is the Euler distance between $s_{j}$ and $q_{i}$.

Based on Equation (1), the received power of $s_{j}$ from the UAV is expressed as

(2) $$P_{q_{i},s_{j}}^{T}=\zeta *h_{j}^{i}*P_{R},$$

where ζ∈(0,1) is the RF-to-DC energy conversion efficiency [19] of sensor $s_{j}$.

### 3.3. Laser Charging Model

In this paper, an MUV equipped with a laser transmitter is used to recharge the UAV. We used the linear energy-harvesting model proposed in [20]. Therefore, the received power of the UAV from the MUV when the UAV is hovering at point $g_{i}$ can be expressed as

(3) $$P_{L}^{i}=\eta *e^{-\ell *d(f,g_{i})}*P_{L},$$

where η∈(0,1) represents the conversion efficiency of laser to electricity, *ℓ* denotes the laser attenuation coefficient, and $d(f,g_{i})$ is the distance between *f* and $g_{i}$. $\ell =\frac{\vartheta_{0}}{\mu }{\left(\frac{\partial }{\vartheta_{1}}\right)}^{-\varsigma }$, where $\vartheta_{0}$ and $\vartheta_{1}$ are two constants, μ represents the visibility of the environment, *∂* is the wavelength, and ς denotes the size distribution of the scattered particles.

### 3.4. Power Consumption Model

In addition to charging the sensors, the UAV also needs energy for operations such as flying and hovering. In this paper, we use the rotary-wing UAV propulsive power consumption model proposed in [11]. Therefore, the consumed power for the UAV flying at the speed *v* can be described as

(4) $$P_{f}=P_{0}\left(1+\frac{3v^{2}}{U_{tip}^{2}}\right)+P_{1}{\left(\sqrt{1+\frac{v^{4}}{4v_{0}^{4}}}-\frac{v^{2}}{2v_{0}^{2}}\right)}^{\frac{1}{2}}+\frac{d_{0}\rho sv^{3}A}{2},$$

where $P_{0}$ and $P_{1}$ are two constants linked to the physical parameters of the UAV and flight environment, $U_{tip}$ represents the tip speed of the rotor blade, $v_{0}$ denotes the average rotor-induced velocity while hovering, $d_{0}$ is the fuselage drag ratio, *s* denotes the rotor stiffness, ρ is the air density, and *A* represents the rotor disk area.

Since v=0 when the UAV is hovering, we bring v=0 into Equation (4) and the power consumption of the UAV can be expressed as

(5) $$P_{h}=P_{0}+P_{1}.$$

### 3.5. Definition of the Problem

Suppose $F(U,Q,T_{Q},G,T_{G},T_{W})$ denotes a feasible flight trajectory of a UAV and travel plans of an MUV such that all the sensors of the network can be charged by a UAV, where *U* is a closed continuous flight trajectory of a UAV that starts and ends at $s_{0}$, *Q* denotes a set of hovering points of a UAV on *U* charging the sensors, *G* represents a set of hovering points of a UAV on *U* recharged by an MUV, $T_{Q}$ and $T_{G}$ are the sets of hovering times for the points in *Q* and *G*, respectively, $T_{W}$ represents the set of hovering times of a UAV waiting for an MUV arriving at the points in *G*. For any hovering point $q_{i}\in Q$ of a UAV, there exists a corresponding hovering time $t_{q}^{i}\in T_{Q}$, which is used to charge the sensors. In addition, for any $g_{j}\in G$, there exists a corresponding hovering time $t_{g}^{j}\in T_{G}$ to replenish the energy of a UAV. Each $t_{w}^{j}\in T_{W}$ denotes the waiting time of a UAV for an MUV hovering at $g_{j}$.

In this paper, our goal is to find the optimal flight trajectory of a UAV and travel plans of an MUV $F(U,Q,T_{Q},G,T_{G},T_{W})$ cooperatively so that the time consumption $T=\frac{L(U)}{v_{f}}+\sum_{t_{q}^{i}\in T_{Q}}t_{q}^{i}+\sum_{t_{g}^{j}\in T_{G}}t_{g}^{j}+\sum_{t_{w}^{j}\in T_{W}}t_{w}^{j}$ is minimized. More formally, we define the research problem as the trajectory optimization of laser-charged UAVs for charging WRSNs (TOLC), as shown in Definition 1.

**Definition** **1** (TOLC). *Given a set $S=\{s_{1},s_{2},...,s_{n}\}$ of n sensors in which each $s_{i}$ stores $E_{s}^{i}$ energy, a UAV with $n_{0}$ antennas, flight speed $v_{f}$, flight height H, initial energy $E_{v}$ and initial location $s_{0}$, an MUV traveling at a defined route L with maximum speed $v_{max}$, the aim of the trajectory optimization of laser-charged UAVs for charging WRSNs (TOLC) is to find the flight trajectory of a UAV and the travel plans of an MUV $F(U,Q,T_{Q},G,T_{G},T_{W})$ such that*

*(1) the tour U starts from and ends at $s_{0}$,*

*(2) the UAV can simultaneously charge at most $n_{0}$ sensors when it hovers at any point $q_{i}\in Q$ with $t_{q}^{i}\in T_{Q}$ time,*

*(3) the energy of the UAV is always greater than or equal to zero and less than or equal to $E_{v}$,*

*(4) the time that the UAV charges the sensors at $q_{i}\in Q$ is $t_{q}^{i}\in T_{Q}$, the time that the MUV recharges the UAV at $g_{i}\in G$ is $t_{g}^{i}\in T_{G}$, and the time for the UAV to wait for the MUV at $g_{j}\in G$ is $t_{w}^{j}\in T_{W}$,*

*(5) the time consumption of the UAV, $T=\frac{L(U)}{v_{f}}+\sum_{t_{q}^{i}\in T_{Q}}t_{q}^{i}+\sum_{t_{g}^{j}\in T_{G}}t_{g}^{j}+\sum_{t_{w}^{j}\in T_{W}}t_{w}^{j}$, is minimized.*

**Theorem** **1.** *The TOLC problem is NP-hard.*

**Proof**  (Proof of Theorem 1). We consider a special case of the TOLC problem where we set $E_{s}^{i}=E_{0}$ for each sensor $s_{i}\in S$, $E_{v}=+\infty$, H=0, $n_{0}=1$ and then the TOLC problem can be reduced to the well-known traveling salesman problem (TSP), where the UAV only needs to visit all sensors located in the detection area. Since the TSP problem is proved NP-hard and it is a special case of the TOLC problem, the TOLC problem is NP-hard. □

## 4. Algorithm for the TOLC Problem

In this section, we propose an approximate algorithm to solve the TOLC problem, which is called the TOLCA. The algorithm consists of two steps. The first step is to find the hovering point set *Q* and corresponding time set $T_{Q}$ of the UAV using the proposed clustering algorithm WCMC so that the UAV can be used to charge all sensors when it is hovering at the points in *Q*. In the second step, we design the flight path of the UAV using the proposed TOLCA algorithm based on *Q* obtained in the first step, which consists of the flight path *U*, charging point set *G* with corresponding time set $T_{G}$, and the waiting time set $T_{W}$ of the MUV.

Therefore, we propose the following WCMC and TOLCA algorithms that correspond to the above steps.

### 4.1. Algorithm Description for WCMC

In this subsection, we propose the weighted centered minimum coverage (WCMC) algorithm to compute the hover positions of the UAV for charging sensors. For simplicity, we use *m* to record the number of clusters and we initially set m=0.

In the first step, we model the WRSN to be an undirected complete graph $G^{\prime }=\left(V^{\prime },E^{\prime }\right)$, where $V^{\prime }=\left\{s_{1},s_{2},...,s_{n}\right\}$ and $E^{\prime }=\left\{\left(s_{i},s_{j}\right)|1\leq i<j\leq n\right\}$. The algorithm repeats the following steps until $E^{\prime }=\emptyset$.

- Select the shortest edge $e_{i,j}$ in the graph *G* such that $d\left(s_{i},s_{j}\right)=min\left\{d\left(s_{u},s_{v}\right)|e_{u,v}\in E^{\prime }\right\}$. If $d(s_{i},s_{j})>r$, then $E^{\prime }=\emptyset$.;
- Calculate the pseudo-center $q_{m}$ of the points $s_{i}$,$s_{j}$ using the algorithm proposed in [21] and let $R_{ij}^{m}=\max\left\{d\left(s_{i},q_{m}\right),d\left(s_{j},q_{m}\right)\right\}$.
- Initialize $Q_{c}$ by comparing $R_{ij}^{m}$ and *r*. If $R_{ij}^{m}\leq r$, then $q_{m}$ is added to $Q_{c}$ and m=m+1. The vertices $s_{i},s_{j}$ are removed from $V^{\prime }$ and all edges adjacent to $s_{i},s_{j}$ are also deleted from $E^{\prime }$.

In the second step, we set $Q_{c}^{o}=\emptyset$, which is a parameter for recording the change in $Q_{c}$. The algorithm repeats the following three steps until $Q_{c}\neq Q_{c}^{o}$.

- For any 1≤j≤n, each sensor $s_{j}$ selects the nearest cluster $\omega (q_{i})$ such that $d\left(s_{j},q_{i}\right)=min\left\{d\left(s_{u},q_{v}\right)|q_{v}\in Q_{c}\right\}$, $\omega \left(q_{i}\right)=\omega \left(q_{i}\right)\cup \left\{s_{j}\right\}$.
- For 1≤i≤m, 1≤j≤m, if $d(q_{i},q_{j})\leq 2r$ and $Num\left(\omega \left(q_{i}\right)\cup \omega \left(q_{j}\right)\right)\leq n_{0}$, then we calculate the weighted center $q_{k}$ of the points in $\omega \left(q_{i}\right)\cup \omega \left(q_{j}\right)$ using the algorithm proposed in [22], where Num({∗}) denotes the number of sensors in {∗}. Afterward, we calculate the disk that is centered at $q_{k}$ and whose radius is $R_{k}^{m}=max\left\{d\left(q_{k},s\right)|s\in \left\{\omega \left(q_{i}\right),\omega \left(q_{j}\right)\right\}\right\}$. If $R_{k}^{m}\leq r$, then we merge $\omega (q_{i})$ and $\omega (q_{j})$ since they are located on the disk.
- For any 1≤i≤m, we calculate the disk that is centered at $q_{i}$ and whose radius is $R_{i}^{m}=max\left\{d\left(q_{i},s_{j}\right)|s_{n}\in \left\{\omega \left(q_{i}\right)\right\}\right\}$. If $R_{i}^{m}>r$ or $Num\left(\omega \left(q_{i}\right)\right)>n_{0}$, then we split $\omega (q_{i})$ since it exceeds the radius of the coverage disk of the UAV or exceeds the maximum number of sensors that the UAV can charge simultaneously. We first calculate the centers $c_{i1},c_{i2}$ as $c_{i1}=\left(x_{min},\frac{y_{max}+y_{min}}{2}\right)$, and $c_{i2}=\left(x_{max},\frac{y_{max}+y_{min}}{2}\right)$, where $x_{min},y_{min}$ and $x_{max},y_{max}$ represent the minimum and maximum values of all sensor coordinates $x_{i}$ and $y_{i}$ in the cluster $\omega (q_{i})$, respectively. Then, for any $1\leq j\leq Num(\omega \left(q_{i}\right))$, if $d\left(s_{j},c_{i1}\right)>d\left(s_{j},c_{i2}\right)$, we assign the sensor $s_{j}$ to the new cluster $\omega (q_{m+1})$. Afterward, we recalculate the weighted center $q_{i}$ and $q_{m+1}$ of the points in $\omega (q_{i})$ and $\omega (q_{m+1})$, respectively, using the algorithm proposed in [22].

Finally, for any 1≤i≤m, we calculate the time consumption $t_{q}^{i}$ and energy consumption $E_{q_{i}}^{R}$ of the UAV charging the sensors in the cluster $Q=\{\omega \left(q_{1}\right),\omega \left(q_{2}\right),\ldots ,\omega \left(q_{m}\right)\}$ at $Q_{c}=\left\{q_{1},q_{2},...,q_{m}\right\}$.

The pseudo-code of the WCMC algorithm is given in Algorithm Table 1.

### 4.2. Algorithm Description for TOLCA

In this subsection, we propose an approximation algorithm for solving the TOLC problem, which is called the TOLCA. The objective of the algorithm is to optimize the flight trajectory of a UAV and the travel plans of an MUV $F(U,Q,T_{Q},G,T_{G},T_{W})$ cooperatively such that the total time consumption of a UAV

$$T=\frac{L(U)}{v_{f}}+\sum_{t_{q}^{i}\in T_{Q}}t_{q}^{i}+\sum_{t_{g}^{j}\in T_{G}}t_{g}^{j}+\sum_{t_{w}^{j}\in T_{W}}t_{w}^{j}$$

is minimized.

The TOLCA algorithm consists of four steps as follows.

In the first step, we divide the WRSN into *m* clusters $Q=\{\omega \left(q_{1}\right),\omega \left(q_{2}\right),\ldots ,\omega \left(q_{m}\right)\}$ using Algorithm 1, where $Q_{c}=\left\{q_{1},q_{2},\ldots ,q_{m}\right\}$ are the hovering centers of the UAV in cluster set *Q*, and we compute the time consumption $T_{Q}=\left\{t_{q}^{1},t_{q}^{2},\ldots ,t_{q}^{m}\right\}$ and the energy consumption $E_{Q}=\left\{E_{q_{i}}^{R},E_{q_{i}}^{R},\ldots ,E_{q_{i}}^{R}\right\}$ for transmitting energy to the sensors of the UAV in each cluster.

**Algorithm 1:** WCMC

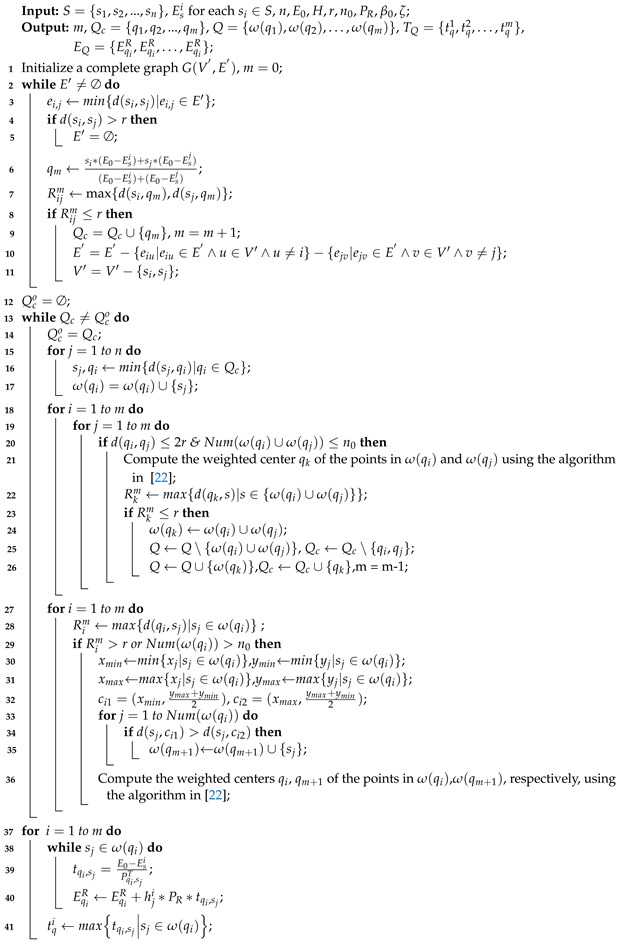



In the second step, we use the genetic algorithm for the TSP problem proposed in [23] to compute a tour $\sigma_{f}$ for $Q_{c}\cup s_{0}$, and obtain the order of points in $Q_{c}$ of the UAV, which is denoted as $q_{\rho_{0}},q_{\rho_{1}},q_{\rho_{2}},...,q_{\rho_{m}}$, where $q_{\rho_{0}}=s_{0}$, and $q_{\rho_{i}}$ is the *i*-th visited by the UAV.

In the third step, for any *i* from 1 to *m*, we calculate the set $C=\{c_{1},c_{2},\ldots ,c_{m}\}$ of points nearest to the hovering points in $Q_{c}$ of the UAV on the *L*.

In the fourth step, we first calculate the remaining energy *E* of the UAV from $q_{\rho_{0}}$ to $q_{\rho_{1}}$ as $E=E-\frac{d(U_{k},q_{\rho_{1}})}{v_{f}}*P_{f}$. Then, for any *i* from 1 to *m*, we calculate the waiting time $t_{w}^{c_{i}}$ of the UAV for the MUV to arrive at $c_{i}$ after charging the sensors at $q_{\rho_{i}}$ as $t_{w}^{c_{i}}=\frac{L(g_{k-1},c_{i})}{v_{\max}}-\frac{d(g_{k-1},\rho_{i},c_{i})}{v_{f}}-t_{\rho }^{i}$, and the waiting time $t_{w}^{\rho_{i+1},c_{i+1}}$ of the UAV for the MUV to arrive at $c_{i+1}$ after charging the sensors at $q_{\rho_{i}}$ and $q_{\rho_{i+1}}$ as $t_{w}^{\rho_{i+1},c_{i+1}}=\frac{L(g_{k-1},c_{i+1})}{v_{max}}-t_{\rho }^{i}-t_{\rho }^{i+1}-\frac{d(g_{k-1},\rho_{i},\rho_{i+1},c_{i+1})}{v_{f}}$; if $t_{w}^{i}\leq 0$ or $t_{w}^{\rho_{i+1},c_{i+1}}<0$, it means that the MUV can reach $c_{i}$ or $c_{i+1}$ when the UAV arrives, then let $t_{w}^{i}=0$ or $t_{w}^{\rho_{i+1},c_{i+1}}=0$ and the corresponding energy consumption $E_{c_{i}}$, $E_{c_{i+1}}$ can be represented as $E_{c_{i}}=E_{q_{\rho_{i}}}^{R}+t_{q}^{i}*P_{h}+\frac{d(q_{\rho_{i}},c_{i})}{v}*P_{f}+t_{w}^{c_{i}}*P_{h}$, $E_{c_{i+1}}=\frac{d(q_{\rho_{i}},q_{\rho_{i+1}},c_{i+1})}{v_{f}}*P_{f}+E_{q_{\rho_{i}}}^{R}+E_{q_{\rho_{i+1}}}^{R}+\left(t_{q}^{i}+t_{q}^{i+1}+t_{w}^{\rho_{i+1},c_{i+1}}\right)*P_{h}$. Then, we select the flight trajectory of the UAV from the following three cases by comparing $E_{c_{i}}$ and $E_{c_{i+1}}$ with the current remaining energy *E* of the UAV.

1. $E\leq E_{c_{i}}$. The remaining energy *E* of the UAV arriving at $q_{\rho_{i}}$ is not enough to charge the sensors at $q_{\rho_{i}}$ and fly to $c_{i}$ for waiting for the MUV to replenish energy. Therefore, the UAV needs to fly to $c_{i}$ to replenish energy and returns to $\omega (q_{\rho_{i}})$ to complete the remaining tasks. Then, we compute $T=T+2*\frac{d(q_{\rho_{i}},q_{c_{i}})}{v_{f}}+\frac{E_{u}}{P_{L}^{i}}+t_{q}^{i}$ and $E=E+E_{u}-\frac{d(q_{\rho_{i}},q_{c_{i}})}{v_{f}}*P_{f}-E_{c_{i}}$ and let $g_{k}=c_{i}$.
2. $E_{c_{i}}\leq E<E_{c_{i+1}}$. The remaining energy *E* of the UAV arriving at $q_{\rho_{i}}$ is enough to charge the sensors at $q_{\rho_{i}}$ and fly to $c_{i}$ to replenish energy but is not enough to reach $q_{\rho_{i+1}}$ for charging the sensors at $q_{\rho_{i}}$, $q_{\rho_{i+1}}$ and fly to $c_{i+1}$ for waiting for the MUV to arrive.
  - Compute whether to have $b_{i}\in B$ on the route *L*, which is the closest point to the UAV path $q_{\rho_{i}}$ to $q_{\rho_{i+1}}$.
  - Calculate the time $t_{w}^{b_{i}}$ for the UAV to wait for the MUV to arrive as $t_{w}^{b_{i}}=\frac{L(g_{k-1},b_{i})}{v_{max}}-\frac{d\left(g_{k-1},q_{\rho_{i}}\right)+d\left(q_{\rho_{i}},b_{i}\right)}{v_{f}}-t_{\rho }^{i}$, if $t_{w}^{b_{i}}<0$, let $t_{w}^{b_{i}}=0$, then compute the required energy $E_{b_{i}}$ for UAV from $q_{\rho_{i}}$ to $b_{i}$ as $E_{b_{i}}=E_{q_{\rho_{i}}}^{R}+t_{q}^{i}*P_{h}+\frac{d(q_{\rho_{i}},b_{i})}{v_{f}}*P_{f}+t_{w}^{b_{i}}*P_{h}$, if $E\geq E_{b_{i}}$, calculate the time $t_{b_{i}}$ for the UAV to fly to $b_{i}$ and then to $q_{\rho_{i+1}}$ as $t_{b_{i}}=\frac{E_{u}-\left(E-E_{q_{\rho_{i}}}^{R}-\left(t_{q}^{i}+t_{w}^{b_{i}}\right)*P_{h}-\frac{d(q_{\rho_{i}},b_{i})}{v_{f}}*P_{f}\right)}{P_{L}^{i}}+\frac{d(q_{\rho_{i}},c_{i+1},q_{\rho_{i+1}})}{v_{f}}$.
  - Calculate the time $t_{w}^{c_{i+1}}$ for the UAV to wait for the MUV to arrive as $t_{w}^{c_{i+1}}=\frac{L(g_{k-1},c_{i+1})}{v_{max}}-\frac{d\left(g_{k-1},q_{\rho_{i}}\right)+d\left(q_{\rho_{i}},c_{i+1}\right)}{v_{f}}-t_{\rho }^{i}$, if $t_{w}^{c_{i+1}}<0$, let $t_{w}^{c_{i+1}}=0$, then compute the required energy $E_{c_{i+1}}$ for the UAV from $q_{\rho_{i}}$ to $c_{i+1}$ as $E_{c_{i+1}}=E_{q_{\rho_{i}}}^{R}+t_{q}^{i}*P_{h}+\frac{d(q_{\rho_{i}},c_{i+1})}{v_{f}}*P_{f}+t_{w}^{c_{i+1}}*P_{h}$. If $E\geq E_{c_{i+1}}$, calculate the time $t_{c_{i+1}}$ of the UAV to fly to $c_{i+1}$ and then to $q_{\rho_{i+1}}$ as $t_{c_{i+1}}=\frac{E_{u}-\left(E-E_{q_{\rho_{i}}}^{R}-\left(t_{q}^{i}+t_{w}^{c_{i+1}}\right)*P_{h}-\frac{d(q_{\rho_{i}},c_{i+1})}{v_{f}}*P_{f}\right)}{P_{L}^{i}}$.
  - Calculate the time $t_{c_{i}}$ for the UAV to fly to $c_{i}$ and then to $q_{\rho_{i+1}}$ as $t_{c_{i}}=\frac{d(q_{\rho_{i}},c_{i},q_{\rho_{i+1}})}{v_{f}}+\frac{E_{u}-\left(E-E_{q_{\rho_{i}}}^{R}-\left(t_{q}^{i}+t_{w}^{c_{i}}\right)*P_{h}-\frac{d(q_{\rho_{i}},c_{i})}{v_{f}}*P_{f}\right)}{P_{L}^{i}}$. Then, compare the time consumptions $t_{c_{i}},t_{b_{i}}$, $t_{c_{i+1}}$ of the UAV and select the point $g_{k}$ with the shortest time $t_{g_{k}}=min\{t_{c_{i}},t_{b_{i}},$$t_{c_{i+1}}\}$ among the points $c_{i},b_{i},c_{i+1}$, and $g_{k}$ as the point where the MUV supplements the power of the UAV. Compute the total time *T* and remaining energy *E* of the UAV to reach $q_{\rho_{i+1}}$, which are expressed as $T=T+t_{g_{k}}+t_{q}^{i}+t_{w}$, $E=E_{u}-\frac{d(g_{k},q_{\rho_{i+1}})}{v_{f}}*P_{f}$.
3. $E\geq E_{c_{i+1}}$. The residual energy *E* of the UAV arriving at $q_{\rho_{i}}$ is sufficient for the UAV to fly from $q_{\rho_{i}}$ to $q_{\rho_{i+1}}$ and charge the sensors at $q_{\rho_{i}}$ and $q_{\rho_{i+1}}$ and fly to $c_{i+1}$ to wait for the MUV to arrive so the UAV can fly directly from $q_{\rho_{i}}$ to $q_{\rho_{i+1}}$. Then, we compute the total time *T* and remaining energy *E* to reach $q_{\rho_{i+1}}$ as $T=T+t_{\rho }^{i}+\frac{d(q_{\rho_{i}},q_{\rho_{i+1}})}{v_{f}}$, $E=E-E_{q_{i}}^{R}-\frac{d(q_{\rho_{i}},q_{\rho_{i+1}})}{v_{f}}*P_{f}$.

Consequently, we can obtain the flight trajectory of the UAV and the travel plans of the MUV $F(U,Q,T_{Q},G,T_{G},T_{W})$ and the total time consumption *T* of the UAV; the pseudo-code of the TOLCA algorithm is given in Algorithm 2.

**Algorithm 2:** TOLCA

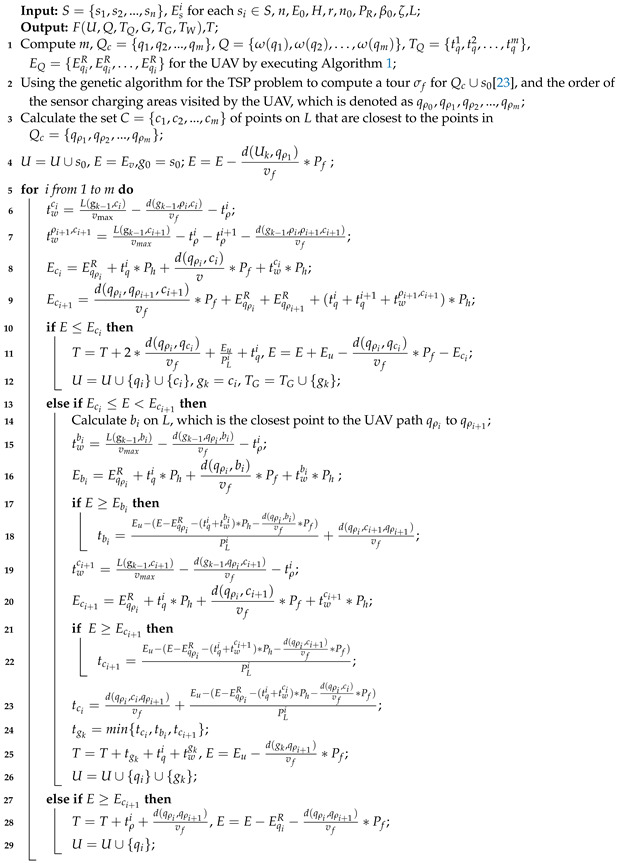



## 5. Simulation Results

In this section, we use MATLAB 2021 to program and evaluate the average performance of the approximation algorithm TOLCA by simulating several key indicators under different settings. Note that in reality, the road of the MUV is very tortuous and irregular, which brings difficulties to modeling and measurement, so we set the trajectory of the MUV *L* as an elliptical $\frac{{\left(x-1000\right)}^{2}}{1000^{2}}+\frac{{\left(y-1000\right)}^{2}}{700^{2}}=1$ to verify the experiment. In the simulation, the sensors are randomly deployed in a 2000 m × 2000 m detection area. Some of the basic experimental parameters are summarized in Table 1, and the average of multiple results is used in the experiments.

**Figure 2:**
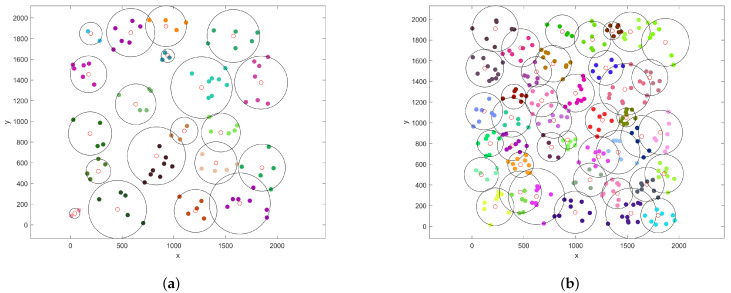

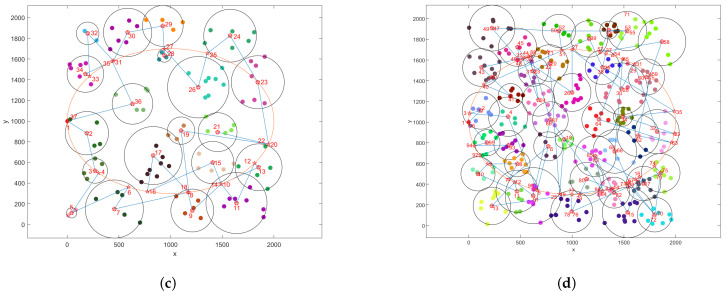
Simulation results for the network. (**a**) The clustering results for the WCMC algorithm when n=100; (**b**) The clustering results for the WCMC algorithm when n=300; (**c**) The planning results for the TOLCA algorithm when n=100; (**d**) The planning results for the TOLCA algorithm when n=300. In Figures (**a**–**d**), the different colored dots represent sensors, the red circles denote the cluster centers, the red diamonds denotes the base station, the red five-points stars represent the hovering positions of UAV, the red numbers represent the order of hovering points visited by UAV, the orange ellipses and blue broken lines denote the trajectories of the MUV and the UAV, respectively.

Figure 2 shows the simulation results obtained using the WCMC and TOLCA algorithms when we set $n_{0}=10$, $v_{f}$ = 20 m/s, $v_{max}$ = 10 m/s, $P_{R}$ = 200 W, $P_{L}$ = 300 W, $E_{v}$ = 10,000 J, r=300 m, and $E_{0}$ = 20 J. Figure 2a,b show that clustering results were obtained using the WCMC algorithm when the number of sensors was 100 and 300, respectively. Figure 2c,d show that the flight trajectories of the UAV were obtained using the TOLCA algorithm when the number of sensors was 100 and 300, respectively.

### 5.1. Comparison Results

**Figure 3:**
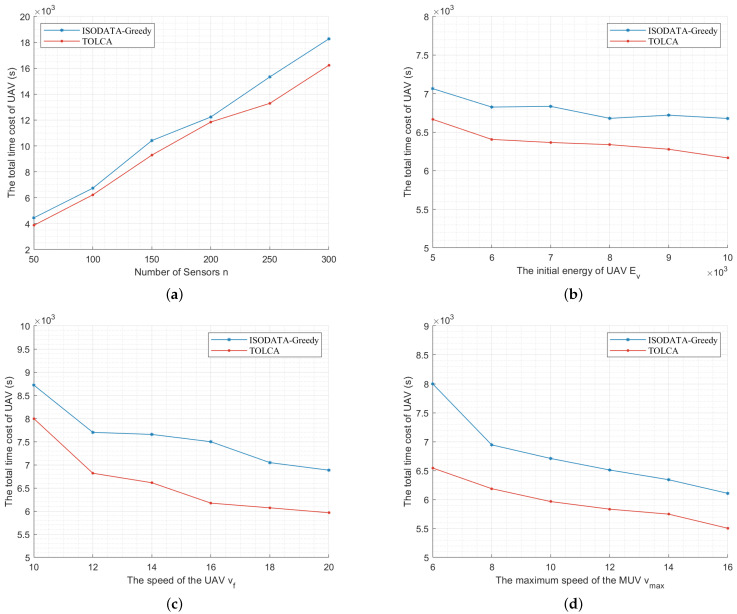

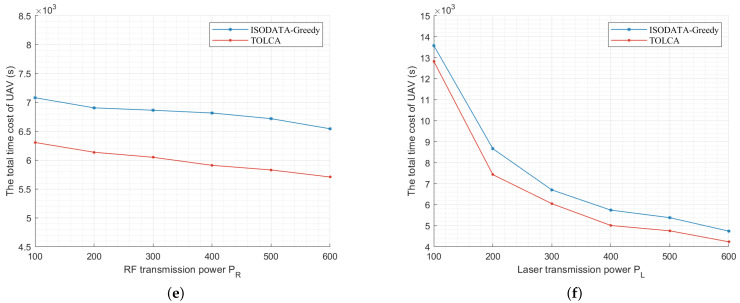
The comparison results between the TOLCA and ISODATA–Greedy algorithms. (**a**) Increasing *n* from 50 to 300; (**b**) Increasing $E_{v}$ from 5000 to 10,000 J; (**c**) Increasing $v_{f}$ from 10 to 20 m/s; (**d**) Increasing $v_{max}$ from 6 to 16 m/s; (**e**) Increasing $P_{R}$ from 100 to 600 W; (**f**) Increasing $P_{L}$ from 100 to 600 W.

In order to prove the effectiveness of the TOLCA algorithm, we propose to combine the clustering algorithm ISODATA and the Greedy algorithm to calculate the total time for the UAV to charge the sensors in the WRSN, as shown in Figure 3. In the greedy algorithm, we first cluster the WRSN using the ISODATA algorithm. Then, we use the genetic algorithm for the TSP problem proposed in [23] to compute the order of the UAV visiting the hovering positions in the clusters. Next, the set of points on the route *L* of the MUV closest to the hovering position of the UAV in the cluster is found. Finally, the remaining energy of the UAV to the hovering point of each cluster is judged; if the remaining energy of the UAV is enough to complete the task in the current cluster and the next cluster and fly to the nearest charging point of the next cluster, it flies directly to the next cluster; otherwise, it needs to replenish the power at the nearest charging point to the current cluster.

Figure 3a shows the total time consumed by the UAV of the two algorithms when we set $n_{0}=10$, $v_{f}=20$ m/s, $v_{max}=10$ m/s, $E_{v}=10,000$ J, r=300 m, $E_{0}=20$ J, $P_{R}=200$ W, $P_{L}=300$ W, and n=50,100,150,200,250,300. We can see that the total time cost of the UAV is approximately proportional to the number of sensors since the time spent charging the sensors increases with the number of sensors. We can also see that our proposal consumes less time than the ISODATA–Greedy algorithm.

Figure 3b shows the simulation results of the two algorithms when we set n=100, $n_{0}=10$, $v_{f}=20$ m/s, $v_{max}=10$ m/s, r=300 m, $E_{0}=20$ J, $P_{R}=200$ W, and $P_{L}=300$ W and vary the UAV’s initial energy $E_{v}$ from 5000 J to 10,000 and J increases by 1000. We can see that the total time of the UAV decreases as $E_{v}$ grows, and we can also see that the time consumption of the UAV is shorter with the TOLCA algorithm, which validates the TOLCA algorithm.

Figure 3c shows the simulation results of the two algorithms when we set n=100, $n_{0}=10$, $v_{max}=10$ m/s, $E_{v}=10,000$ J, r=300 m, $E_{0}=20$ J, $P_{R}=200$ W, and $P_{L}=300$ W and change the UAV’s speed $v_{f}$ from 10 m/s to 20 m/s. We can see that the total time of the UAV decreases with the increase in $v_{f}$ because the time consumed by the UAV during the flight becomes shorter. We can also see that the time consumed by the UAV with the TOLCA algorithm decreases as the speed of the UAV increases, proving that our proposed algorithm produced better results.

Figure 3d gives the simulation results of these two algorithms when we set n=100, $n_{0}=10$, $v_{f}=20$ m/s, $E_{v}=\;10,000$ J, r=300 m, $E_{0}=\;20$ J, $P_{R}=200$ W, and $P_{L}=300$ and vary $v_{max}$ from 6 m/s to 16 m/s. We can see that the total time of the UAV decreases with the increase in $v_{max}$. This is because as the speed of the MUV increases, the time that the UAV waits for the MUV to arrive becomes shorter. We can also see that the total time spent by the UAV with the TOLCA algorithm is shorter.

Figure 3e gives the simulation results of the two algorithms when we set n=100, $n_{0}=10$, $v_{f}=20$ m/s, $v_{max}=10$ m/s, $E_{v}=10,000$ J, r=300 m, $E_{0}=\;20$ J, and $P_{L}=300$ W and change $P_{R}$ from 100 W to 600 W. The results show that the TOLCA algorithm outperforms the combined ISODATA–Greedy algorithms. We can also see that the time cost of the UAV obtained using the two algorithms decreases monotonically with an increasing $P_{R}$ since the time for the UAV to charge the sensors decreases as $P_{R}$ increases.

Figure 3f gives the simulation results of the two algorithms when we set n=100, $n_{0}=10$, $v_{f}=20$ m/s, $v_{max}=10$ m/s, $E_{v}=10,000$ J, r=300 m, $E_{0}=20$ J, and $P_{R}=200$ W and vary $P_{L}$ from 100 W to 600 W. The results show that the TOLCA algorithm outperforms the combined ISODATA–Greedy algorithms. We can also see that the time cost of the UAV obtained by the two algorithms decreases with an increasing $P_{L}$ since the time for the MUV to charge the UAV decreases with an increasing $P_{L}$.

### 5.2. Impact of Network Configurations with TOLCA

In the following, we evaluate the impact of the different parameter settings on the total time cost of the UAV.

**Figure 4:**
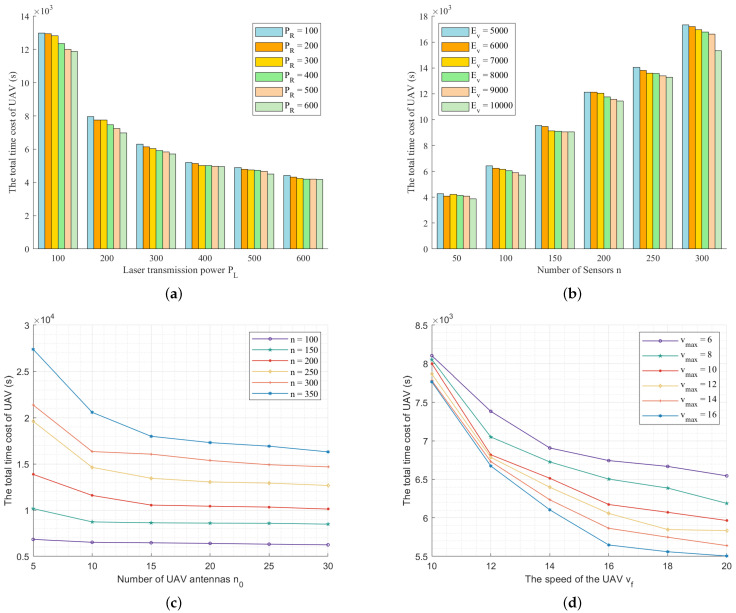

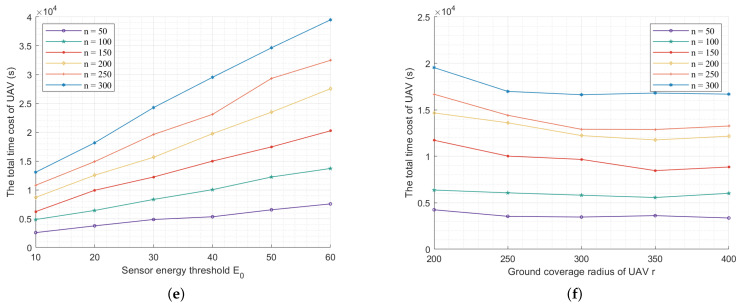
The performance of TOLCA under different configurations. (**a**) Increasing $P_{L}$ from 100 to 600 W; (**b**) Increasing *n* from 50 to 300; (**c**) Increasing $n_{0}$ from 5 to 30; (**d**) Increasing $v_{f}$ from 10 to 20 m/s; (**e**) Increasing $E_{0}$ from 10 to 60 J; (**f**) Increasing *r* from 200 to 400 m.

Figure 4a illustrates the performance of TOLCA when we set n=100, $n_{0}=10$, $v_{f}=20$ m/s, $v_{max}=10$ m/s, $E_{v}=10,000$ J, r=300 m, $E_{0}=20$ J, and $P_{R}=100,200,300,400,500,600$ and change $P_{L}$ from 100 W to 600 W. We can see that the total time of the UAV decreases as the laser transmission power $P_{L}$ increases. This is because when $P_{L}$ increases, the hovering time of the UAV to replenish energy from the MUV decreases. In addition, we can see that the total time of the UAV decreases with an increasing $P_{R}$ since the hovering time of the UAV for charging the sensors decreases as $P_{R}$ increases.

Figure 4b gives the simulation results when we set $n_{0}=10$, $v_{f}=20$ m/s, $v_{max}=10$ m/s, $P_{R}=200$ W, $P_{L}=300$ W, r=300 m, $E_{0}=\;20$ J, and $E_{v}$ = 5000, 6000, 7000, 8000, 9000, 10,000 and vary the number of sensors *n* from 50 to 300, increased by 50. We can see that the total time of the UAV grows as *n* increases since the time to charge the sensors, the number of times to replenish energy, and the flying distance increase with an increasing *n*. We can also see that the total time of the UAV decreases as $E_{v}$ increases. This is because the number of times for replenishing energy is reduced.

Figure 4c shows the simulation results when we set $v_{f}=20$ m/s, $v_{max}=10$ m/s, $P_{R}=200$ W, $P_{L}=300$ W, $E_{v}=10,000$ J, r=300 m, $E_{0}=\;20$ J, and n=100,150,200,250,300,350 and change $n_{0}$ from 5 to 30, increased by 5. We can see that the total time of the UAV decreases with an increasing $n_{0}$ since the number of sensors that the UAV can cover increases and the flying distance of the UAV decreases. We can also see that the total time of the UAV decreases significantly when $n_{0}$ changes in the large range of the total number of sensors. The greater the number of sensors, the larger $n_{0}$, and the fewer the clusters in the network, the shorter the flight distance of the UAV. When the number of sensors is small, the sensors are dispersed and $n_{0}$ has little influence on the overall network clustering.

In Figure 4d, we illustrate the impact of the UAV and MUV speeds when we set n=100, $n_{0}=10$, $P_{R}=200$ W, $P_{L}=300$ W, $E_{v}$ = 10,000 J, r=300 m, $E_{0}=20$ J, and $v_{max}$ = 6 m/s, 8 m/s, 10 m/s, 12 m/s, 14 m/s, 16 m/s and change $v_{f}$ from 10 m/s to 20 m/s. We can see that the total time of the UAV decreases with an increasing $v_{f}$ since the flying time of the UAV decreases. Meanwhile, we can see that the total time of the UAV decreases with an increasing $v_{max}$ since the hovering time for waiting for the MUV decreases as $v_{max}$ decreases.

In Figure 4e, we evaluate the impact of the sensor energy threshold $E_{0}$ on the time cost of the UAV in the cases where *n* is equal to 50,100,150,200,250,300 when we set $n_{0}=10$, $v_{f}$ = 20 m/s, $v_{max}$ = 10 m/s, $P_{R}=200$ W, $P_{L}=300$ W, $E_{v}$ = 10,000 J, and r=300 m and vary $E_{0}$ from 10 J to 60 J. The results demonstrate that with the sensor energy threshold $E_{0}$ increasing, the total time cost of the UAV increases linearly in these six cases. This is because the hovering time of the UAV to charge the sensors increases as the energy threshold of the sensors increases.

In Figure 4f, we measure the total time cost of the UAV when we set n=50, 100, 150, 200, 250, 300, $n_{0}=10$, $v_{f}=20$ m/s, $v_{max}=10$ m/s, $P_{R}$ = 200 W, $P_{L}$ = 300 W, $E_{v}$ = 10,000 J, and $E_{0}$ = 20 J and vary the ground coverage of the UAV *r* from 200 m to 400 m. We can see that the time cost of the UAV decreases as *r* increases since the traveling time of the UAV decreases with an increasing *r*. We can also see that when the coverage radius of the UAV is too large, the time cost of the UAV gradually increases. This is because as the coverage radius of the UAV increases, the efficiency of the UAV to charge the sensors decreases and the time of the UAV to charge the sensors increases. At the same time, the energy consumption of the UAV increases, the time of the UAV to replenish energy increases, and the time to recharge the UAV increases.

## 6. Conclusions

In this paper, we identify the trajectory optimization of laser-charged UAVs for charging WRSNs (TOLC problem), which focuses on optimizing the flight trajectory of a UAV and the travel plans of an MUV. Then, we prove that the problem is NP-hard. We first propose a clustering algorithm WCMC to cluster the sensors and compute the weighted center of each cluster. Based on the WCMC algorithm, we propose an approximate algorithm TOLCA, which provides not only the flight trajectory, hovering scheme, and corresponding hovering time of the UAV but also the charging points and corresponding charging times of the MUV for charging the UAV. In the simulations, we first compare the results obtained using the proposed algorithm and the ISODATA algorithm combined with the Greedy algorithm to prove the effectiveness of the proposed algorithm. Then, we measure the effectiveness of the TOLCA algorithm by setting different network configurations.

## Figures and Tables

**Table 1:** Experimental parameters.

Notation	Physical Meaning	Value
$\gamma_{0}$	The channel power gain at *d* = 1 m	30
ζ	The RF-to-DC energy conversion efficiency	0.6
*H*	Flight altitude of the UAV	5
η	The conversion efficiency of laser to electricity	0.15
*ℓ*	Laser attenuation coefficient	$10^{-6}$
$P_{0}$	Blade power	14.7517
$P_{1}$	Induced power	41.5409
$U_{tip}$	Tip speed of the rotor blade	80
$v_{0}$	The average rotor-induced velocity	5.0463
$d_{0}$	The fuselage drag ratio	0.5009
ρ	Air density in kg/m${}^{3}$	1.225
*s*	Rotor solidity	0.1248
*A*	Rotor disc area in m${}^{2}$	0.1256

## Data Availability

Not applicable.
